# *EGR3* Immediate Early Gene and the Brain-Derived Neurotrophic Factor in Bipolar Disorder

**DOI:** 10.3389/fnbeh.2018.00015

**Published:** 2018-02-05

**Authors:** Bianca Pfaffenseller, Flavio Kapczinski, Amelia L. Gallitano, Fábio Klamt

**Affiliations:** ^1^Laboratory of Molecular Psychiatry, Hospital de Clínicas de Porto Alegre, Universidade Federal do Rio Grande do Sul, Porto Alegre, Brazil; ^2^Department of Psychiatry and Behavioral Neurosciences, McMaster University, Hamilton, ON, Canada; ^3^Department of Basic Medical Sciences, College of Medicine, University of Arizona, Phoenix, AZ, United States; ^4^Laboratory of Cellular Biochemistry, Department of Biochemistry, Universidade Federal do Rio Grande do Sul, Porto Alegre, Brazil

**Keywords:** immediate early genes, early growth response gene 3 (EGR3), brain-derived neurotrophic factor (BDNF), bipolar disorder, neuroplasticity, regulatory network

## Abstract

Bipolar disorder (BD) is a severe psychiatric illness with a consistent genetic influence, involving complex interactions between numerous genes and environmental factors. Immediate early genes (IEGs) are activated in the brain in response to environmental stimuli, such as stress. The potential to translate environmental stimuli into long-term changes in brain has led to increased interest in a potential role for these genes influencing risk for psychiatric disorders. Our recent finding using network-based approach has shown that the regulatory unit of early growth response gene 3 (*EGR3*) of IEGs family was robustly repressed in postmortem prefrontal cortex of BD patients. As a central transcription factor, EGR3 regulates an array of target genes that mediate critical neurobiological processes such as synaptic plasticity, memory and cognition. Considering that *EGR3* expression is induced by brain-derived neurotrophic factor (BDNF) that has been consistently related to BD pathophysiology, we suggest a link between BDNF and EGR3 and their potential role in BD. A growing body of data from our group and others has shown that peripheral BDNF levels are reduced during mood episodes and also with illness progression. In this same vein, BDNF has been proposed as an important growth factor in the impaired cellular resilience related to BD. Taken together with the fact that EGR3 regulates the expression of the neurotrophin receptor p75NTR and may also indirectly induce BDNF expression, here we propose a feed-forward gene regulatory network involving EGR3 and BDNF and its potential role in BD.

Bipolar disorder (BD) is a chronic and potentially severe and disabling mental illness that affects between 1% and 3% of the population worldwide (Merikangas et al., 2011), and is characterized by episodes of mania and depression. Studies evaluating concordance rates between monozygotic twins indicate that 40%–70% of risk for BD is genetically determined (Kieseppa et al., 2004; Craddock and Sklar, 2013). BD is likely influenced by numerous genes, which may individually contribute only a small risk for the disorder but may interact at the gene-network level and respond to environmental stimuli in a complex interaction.

In addition to the genetic contribution to BD, environment influences (Schmitt et al., 2014; Aldinger and Schulze, 2017) risk through both stressors and protective factors, such as childhood trauma and level of maternal care (Champagne and Curley, 2009; Jansen et al., 2016; Aldinger and Schulze, 2017). The impact that environmental has on the clinical BD course (Aldinger and Schulze, 2017) suggests a potential role for genes that are involved in the response and adaptation to stress. This capacity of immediate early genes (IEGs) to translate environmental stimuli into long-term alterations in the brain makes this class of genes of great interest to the field of psychiatry.

## Immediate Early Genes and Psychiatry

IEGs are a class of genes rapidly and transiently activated in response to a wide range of environmental stimuli (Senba and Ueyama, 1997). Many IEGs encode transcription factors, which regulate downstream target genes that presumably mediate their roles in neurobiological processes including synaptic plasticity and memory formation (Gallitano-Mendel et al., 2007; Poirier et al., 2008; Pérez-Cadahía et al., 2011). Early growth response (EGR) proteins are a family of IEG-encoded transcription factors: EGR1, EGR2, EGR3 and EGR4 (Beckmann and Wilce, 1997; Pérez-Cadahía et al., 2011). EGRs could translate environmental influence into long-term changes in the brain and thus contribute to neuronal plasticity, which has driven to the hypothesis that dysfunction in EGRs may be implicated in both the genetic and environmental involvement on psychiatric disorders susceptibility (Moises et al., 2002; Hanson and Gottesman, 2005; Gallitano et al., 2012; Huentelman et al., 2015).

Studies investigating the potential role of EGR family genes on risk for psychiatric disorders have focused most on the schizophrenia; the most positive findings have been on early growth response gene 3 (*EGR3*). Single nucleotide polymorphisms (SNPs) in *EGR3* are associated with schizophrenia (Kim S. H. et al., 2010; Zhang et al., 2012; Huentelman et al., 2015), and EGR3 mRNA expression is decreased in the postmortem brains of schizophrenia patients compared with controls (Mexal et al., 2005; Yamada et al., 2007). Furthermore, a bioinformatics analysis of the network of transcription factors and microRNAs associated with schizophrenia indicated *EGR3* as a central gene in this regulatory network (Guo et al., 2010).

Regarding a potential role for EGRs in BD, a study focused on association of genes related to circadian rhythms with BD found a nominally significant association for *EGR3* (Mansour et al., 2009). A family-based association study, although limited by small sample size, also showed a nominal and preliminary association of *EGR3* with risk for BD in children (Gallitano et al., 2012), suggesting this gene as a target for subsequent larger follow-up evaluation. Our recent study using a network-based approach showed that the regulatory unit of *EGR3* was robustly reduced in both of the two independent bipolar gene expression signatures examined from postmortem prefrontal cortex (Pfaffenseller et al., 2016), suggesting the entire network centered on *EGR3* might be dysregulated in BD.

Interestingly, *EGR3*-deficient mice, knockout animals generated by targeted mutagenesis in embryonic stem cells (Tourtellotte and Milbrandt, 1998) and thus lacking functional EGR3 in all cells throughout development, show both physiologic and behavioral changes that corroborate with models in psychiatry. Such alterations involve a heightened stress-reactivity (indicated by both an increased behavioral response and elevated corticosterone release following handling, a mild stressor test), hyperactivity in the locomotor activity test (indicating a psychosis-like phenotype), disrupted habituation to environmental stimuli and social cues and increased aggressive behavior toward an unfamiliar animal (Gallitano-Mendel et al., 2007, 2008). These observations suggest that EGR3 may be involved in biological mechanisms needed to an appropriate response to stress that possibly are dysfunctional in BD. Psychosis-like phenotypes and hyperactivity observed in these *EGR3*-deficient mice could be reversed with antipsychotic drugs used in treatment of psychiatric disorders, providing an additional support for these findings (Gallitano-Mendel et al., 2008; Williams et al., 2012).

In this scenario, a recent study showed that EGR3 seems to play an essential role in the susceptibility to stress since it was related to dendritic atrophy in nucleus accumbens medium spiny neurons in mice susceptible to the social defeat stress model, and *EGR3* knockdown inhibited this dendritic atrophy (Francis et al., 2017). The authors suggest that these alterations in dendritic structure mediated by EGR3 could be responsible for loss in the total number of synapses and consequently reduction in the excitatory transmission observed in these defeated mice. This molecular mechanism mediated by EGR3 could reinforce its role in regulating homeostasis and cellular adaptations possibly underlying stress-induced behavior, which highlight the relevance in studying this transcription factor in psychiatry.

## Early Growth Response 3 Pathway

EGR genes are expressed at basal levels throughout the brain, such as the cortex, the hippocampus and the basal ganglia as observed in animal studies (Senba and Ueyama, 1997). EGR3 expression is rapidly induced at high levels in response to environmental alterations, including stressful stimuli and sleep deprivation (Honkaniemi et al., 2000; Thompson et al., 2010; Maple et al., 2015).

The EGR3 expression in neurons is regulated by synaptic activity and is mediated by MAPK-ERK signaling (O’Donovan et al., 1999; Li et al., 2007). Studies have improved the understanding about the signaling cascade that leads to EGR3 expression. EGR3 is induced downstream of numerous proteins, comprising neuregulin 1 (NRG1), calcineurin (CaN), N-methyl-D-aspartate (NMDA) receptors and neurotrophins such as brain-derived neurotrophic factor (BDNF) and nerve growth factor (NGF; Yamagata et al., 1994; Hippenmeyer et al., 2002; Roberts et al., 2006; Yamada et al., 2007; Eldredge et al., 2008).

**Figure 1:**
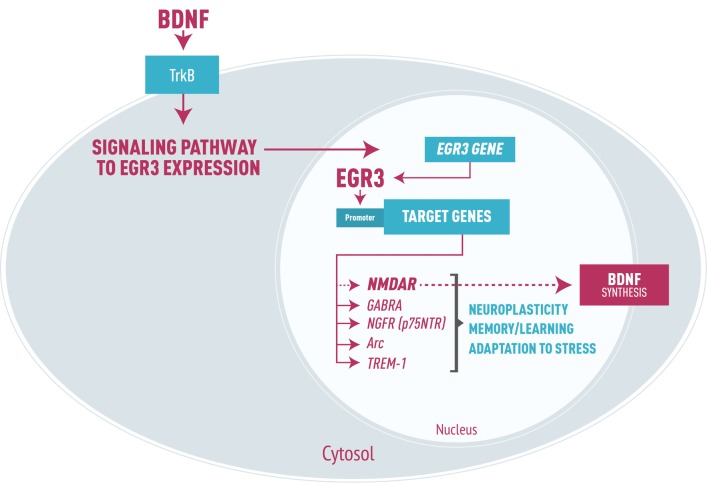
Representation of early growth response gene 3 (EGR3) signaling cascade in neurons, focused on brain-derived neurotrophic factor (BDNF) signaling leading to EGR3 expression. EGR3 is activated downstream of numerous proteins, including BDNF through binding to its receptor TrkB. In turn, EGR3 protein activates numerous downstream target genes. Examples include: type A GABA receptor (GABRA), NGFR (p75NTR) receptor, the activity regulated cytoskeletal associated gene (Arc) and triggering receptor expressed on myeloid cells 1 (TREM-1), as well as, though perhaps indirectly, NMDA receptor (NMDAR). These genes are each involved in critical neurobiological processes such as neuroplasticity, memory and learning and adaptation to stress. It is important to note that EGR3 may indirectly induce BDNF expression via regulation of NMDAR, the activation of which stimulates BDNF synthesis. Thus, we propose a feed-forward regulatory gene network involving EGR3 and BDNF that regulates neuronal gene expression in response to endogenous or environmental stimuli which, when disrupted, may lead to bipolar disorder (BD) pathophysiology.

As a transcription factor, EGR3 could, in turn, activate numerous downstream targets that participate in processes such as synaptic plasticity, axon and dendrites extension and modulation of receptors. Experimental studies have identified effects of EGR3 on NMDA receptors (NMDAR; Gallitano-Mendel et al., 2007), type A gamma amino butyric acid (GABA) receptors (Roberts et al., 2006), and NGF receptors (*p75NTR*, Gao et al., 2007). EGR3 also regulates *Arc* (activity regulated cytoskeletal associated gene; Li et al., 2005), which modifies synapses in response to environmental stimuli, and genes involved in the development and branching of axons and dendrites (Quach et al., 2013). Moreover, it may modulate genes involved in microglia dysregulation such as triggering receptor expressed on myeloid cells 1 (*TREM-1*, Weigelt et al., 2011). Thus, requirement for EGR3 in processes of memory, learning and neuroplasticity (Gallitano-Mendel et al., 2007; Li et al., 2007) is presumably to be determined by these and possibly other EGR3 target genes not yet identified (Figure 1).

## Brain-Derived Neurotrophic Factor and Bipolar Disorder

BDNF is the most highly expressed neurotrophin in the CNS, including brain regions associated with emotion modulation and cognitive processing such as prefrontal cortex, amygdala and hippocampus (Lu et al., 2005, 2014). In addition to its expression in the brain, BDNF is also expressed in peripheral tissues (Fujimura et al., 2002). BDNF plays a critical role in neuronal survival and differentiation, dendritic arborization, synaptic plasticity and also in complex process such as memory consolidation and learning (Minichiello, 2009; Park and Poo, 2013; Lu et al., 2014). BNDF is one of the most extensively investigated biomarkers in BD (Post, 2007; Grande et al., 2010).

Some studies have associated changes in peripheral BDNF levels with BD state, suggesting that serum BDNF levels may represent a potential biomarker of mood episodes. A study by Cunha et al. (2006) reported that patients experiencing an episode of either mania, hypomania, or depression had reduced serum BDNF levels compared to euthymic patients, who had BDNF levels similar to healthy subjects. Another study also showed decreased BDNF levels in transformed lymphoblasts from BD patients in comparison to controls (Tseng et al., 2008). Subsequently, meta-analyses have supported that patients in either a depressive or a manic state have lower blood levels of BDNF than healthy individuals; and serum BDNF levels in euthymic patients did not differ from those observed in controls (Lin, 2009; Fernandes et al., 2011, 2014). A more recent meta-analysis indicated that peripheral BDNF levels are reduced in patients compared to healthy controls, regardless of mood state (Munkholm et al., 2015). Considering central tissue, postmortem studies also have importantly reported alterations in BDNF in BD. For instance, a meta-analysis using postmortem findings from the Stanley Neuropathology Consortium found lower hippocampus BDNF protein levels in BD (Knable et al., 2004). Consistent with this analysis, recent studies have shown that BDNF mRNA was significantly reduced in the hippocampus of BD patients compared to healthy subjects (Thompson Ray et al., 2011; Reinhart et al., 2015). Decreased levels of BDNF have been also found in frontal cortex (protein and mRNA; Kim H. W. et al., 2010) and in inferior and superior temporal gyrus (mRNA) of BD patients (Ray et al., 2014).

Other studies suggest that BDNF may be associated with illness progression. One report showed that BD patients in later stages of the illness show decreased BDNF levels compared to patients in earlier stages, even during euthymic periods (Kauer-Sant’Anna et al., 2009). Moreover, serum BDNF levels are inversely associated with both duration of illness (Kauer-Sant’Anna et al., 2009) and with severity of manic and depressive symptoms (Cunha et al., 2006). Overall the majority of studies indicated that levels of this neurotrophic factor are reduced in bipolar patients compared to controls.

Several studies have been performed to elucidate the mechanisms involved in the presumed reduction in BDNF levels in BD. Some of them have studied polymorphisms in the *BDNF* gene such as the polymorphism involving a methionine substitution for a valine at codon 66 (val66met) in the promoter region of the gene. However, the findings regarding association between the val66met polymorphism and BD are divergent (Sklar et al., 2002; Neves-Pereira et al., 2005), which suggests that this particular variant does not explain the altered BNDF levels in BD (Post, 2007).

The apparent decrease in BDNF levels seen in patients would be expected to result in disruption of the intracellular signaling cascades that are normally regulated by BDNF, such as PLC/PKC, PI3K/Akt and Ras/Erk pathways, interfering with processes regulated by this neurotrophin, such as neuronal differentiation and survival, and synaptic plasticity. In fact, BD has been associated with changes in factors involved in neuroplasticity and resilience pathways, including alterations in apoptotic factors, synaptic markers, neurotrophic and inflammatory factors and oxidative stress markers, as well as in processes related to circadian rhythm, neuronal development and calcium metabolism (Kim H. W. et al., 2010; Frey et al., 2013).

Neuropathological findings in the postmortem brains of BD patients demonstrate the types of abnormalities in neuroplasticity one would expect to see from a deficit in neurotrophic factors. For example, morphometric studies show that patients have enlarged third and lateral ventricles, decreased volume of the orbital and medial prefrontal cortices, ventral striatum and mesotemporal cortex and increased volume of the amygdala compared to controls (Strakowski et al., 2005). Interestingly, such neuroanatomical changes have been found to be more pronounced in patients with multiple mood episodes (Strakowski et al., 2002), suggesting that these abnormalities may increase with severity of the illness (Fornito et al., 2007; Berk et al., 2011). An effective neuroplasticity, considered a cellular and molecular level of adaptation, is likely necessary for the process of resilience, which involves a whole-organism level response to events. Thus, the abnormalities in neuroplasticity possibly translate into reduced resilience related to recurrent mood episodes and illness progression, which could reflect clinically in cognitive impairments in BD patients. In fact, meta-analyses show that most patients exhibit neurocognitive dysfunction, and the most impaired domains are attention, verbal learning, memory and executive functions (Robinson et al., 2006; Bourne et al., 2013; Bortolato et al., 2015).

These findings support the “allostatic load” hypothesis that we have previously described (Kapczinski et al., 2008). This hypothesis asserts that the clinical BD course is determined by a combination of the individual’s genetic makeup, history of stressful life events and degree and duration of episodes of mental illness. These factors are connected in a feedback loop that worsens the patient’s degree of symptoms or overall life function, leading to a progressive illness course associated with biological and brain changes, and cognitive and functioning impairment—hypothesis of BD neuroprogression (Berk, 2009; Fries et al., 2012). Since stress plays an essential role in both the onset and progression of BD, it is noteworthy that BDNF-related neuroplasticity may be a crucial mediator of the effects of stress on BD. Thus, we could assume that a possible dysfunction in neurotrophin pathway might influence an increased vulnerability of BD patients to stressful conditions.

## Link between Early Growth Response 3 and Brain-Derived Neurotrophic Factor in Bipolar Disorder

This perspective article presents an accumulation of findings indicating that changes in BDNF are a consistent feature of BD, and may contribute to the pathophysiology of this mental illness. Here we summarize the potential molecular links between BDNF and the IEG transcription factor EGR3, two molecules that may each play a critical role in the impaired cellular resilience related to BD (Manji et al., 2003; Berk et al., 2011; Pfaffenseller et al., 2016).

As we have discussed, BDNF is reported to be altered in BD in both peripheral and central tissue, and it is possible that blood BDNF levels correlate positively with brain BDNF levels (Klein et al., 2011). Thus, presuming that the reduced peripheral BDNF levels observed in BD patients accurately reflect levels in the brain, and considering that BDNF may induce EGR3 expression via PKC/MAPK dependent pathway (Roberts et al., 2006), the decreased levels of BDNF may account, at least in part, for the prefrontal cortex EGR3 repression that we identified in BD (Pfaffenseller et al., 2016).

Alternatively, or in addition, EGR3 may indirectly induce BDNF expression via regulation of NMDAR. In mice, EGR3 has been shown to be required for the function of NR2B-containing NMDARs (Gallitano-Mendel et al., 2007). A study has shown that the majority of NMDAR protein levels (NR1 subunits) in rat cortical neurons are regulated by the transcription factors CREB and EGR3 (Kim et al., 2012), and NMDAR activation stimulates BDNF synthesis (Marini et al., 1998). It is noteworthy that NMDARs have a critical role for memory processes involving long-term potentiation (LTP) and long-term depression (LTD; Bliss and Collingridge, 1993; Collingridge and Bliss, 1995), and it has been demonstrated that BDNF as well as EGR3 participates in LTP and LTD processes. For example, the administration of exogenous BDNF to mice deficient for BDNF restores the impairment in LTP (Patterson et al., 2001). Mice deficient for *EGR3* have deficits in hippocampal LTD and exhibit dysfunction in NMDAR subclasses (NR1/NR2B; Gallitano-Mendel et al., 2007). Thus, dysfunction in signaling pathways involving both BDNF and EGR3 may underlie the cognitive impairment shown by BD patients.

In addition, EGR3 also regulates the expression of NGFR (*p75NTR*, Gao et al., 2007), a receptor for neurotrophins involved in the control of axonal elongation, neuron survival and differentiation (Dechant and Barde, 2002). Neurogenic potential seems to be mediated by p75(NTR) and is greatly enhanced *in vitro* after treatment with BDNF (Young et al., 2007), indicating this EGR3-regulated mechanism integrate a relevant pathway involved in neuroplasticity that likely correspond to changes in BDNF levels in psychiatric conditions.

Taking this into account, we also suggest that EGR3 repression seen in BD patients could be responsible for the reduced BDNF levels associated to this illness. It is most likely to think in a feedback network than in a cause-and-effect relationship considering that EGR3 is responsive to BDNF and regulates NMDAR, which transcription is also mediated by BDNF through activation of the TrKB receptor and in turn induce BDNF synthesis (Kim et al., 2012). Thus, we propose a feed-forward regulatory gene network involving EGR3 and BDNF (Figure 1) that may regulate biological mechanisms to change neuronal expression according to endogenous or environmental stimuli, and this process might potentially be related to BD pathophysiology.

Altogether, the findings discussed in this article suggest a potential regulatory pathway that possibly is disrupted in BD. For many years, evidence has pointed to alterations in neurotrophic factors in BD suggesting these changes could contribute to an impaired neuroplasticity and resilience. However, the mechanisms underlying this impairment remain unknown. The identified network focused on *EGR3* thus emerges as a potential central player responsible for some of changes observed in BD such as a reduced neurotrophic support. Taking into consideration that EGR3 translates environmental events into neural long-term alterations, the possible disturbance in molecular pathways involving EGR3 could result in an impaired response and adaptation to stress.

We suggest experimental approaches to test the hypothesis regarding a potential feed-forward mechanism involving BDNF and EGR3 and its role in BD, which might contribute to understanding its pathophysiology. Moreover, as a central transcription factor of a gene network that regulates crucial neurobiological processes, EGR3 may be a promising pharmacological target. Modulation of IEGs as *EGR3* might be beneficial since they could provide a dynamic and fast response to neural activity and thus a sustained adaptation through regulation of an entire regulatory gene network.

**Figure 2:**
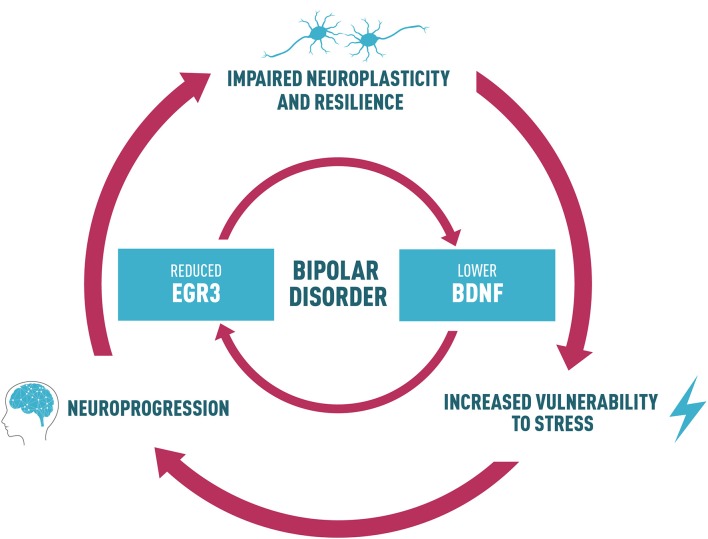
Proposed link between BDNF and EGR3 and their potential role in BD. Lower BDNF levels observed in BD patients may influence the reduced EGR3 levels seen in BD since BDNF regulates EGR3. EGR3 may also indirectly induce BDNF expression via regulation of NMDAR. Thus, we also suggest that reduced EGR3 expression, as we have seen in BD patients in our study, could contribute to lower BDNF levels associated with this illness. Based on these findings, we propose a feedback-loop reinforcing this dysfunctional pathway that could, in turn, impair neuroplasticity and resilience. This process may ultimately lead to increased vulnerability to stress, and could result in alterations in several biological factors that contribute to BD, such as abnormal structural brain changes and the associated cognitive and functional decline (a process called neuroprogression). The neural circuits additionally disrupted in this process could contribute to an impaired neuroplasticity and resilience, increasing vulnerability to stress and mood episodes and reduced responsiveness to pharmacotherapy, thus perpetuating a vicious cycle in illness progression.

With this perspective, we propose that a reduction in EGR3 in BD could contribute to alterations in a neurotrophin cascade in this disorder, which includes reduced BDNF levels. A feedback-loop reinforcing this dysfunctional pathway could, in turn, impair neuroplasticity and resilience (Figure 2). This process may ultimately lead to increased vulnerability to stress, underlying the risk to develop the symptoms and BD neuroprogression. Thus, we suggest an interesting link between EGR3 and BDNF in BD, and this shared biological pathway could provide potential targets for follow-up studies to clarify mechanisms responsible for the interaction between environment and genetic factors that influence BD and for the development of novel therapeutics.

## Author Contributions

All authors provided substantial contributions to the work. BP, FKa and FKl participated in the article design and outline. BP wrote the first draft. ALG revised it critically for relevant scientific content. After a few revisions and editing by all authors, the article was submitted.

## Conflict of Interest Statement

The authors declare that the research was conducted in the absence of any commercial or financial relationships that could be construed as a potential conflict of interest.
